# Migrasomes, new vescicles as Hansel and Gretel white pebbles?

**DOI:** 10.1186/s13062-022-00321-1

**Published:** 2022-04-28

**Authors:** Arianna Di Daniele, Ylenia Antonucci, Silvia Campello

**Affiliations:** Department of Biology, University of Rome “Tor Vergata”, Rome, Italy

**Keywords:** Migrasome, Cell migration, Communication, Signalling, Extracellular vesicles

## Abstract

Migrasomes, released by migrating cells, belong to the heterogeneous world of extracellular vesicles (EVs). However, they can be distinguished from all other members of EVs by their size, biorigin and protein cargo. As far as we know, they can play important roles in various communication processes, by mediating the release of signals, such as mRNAs, proteins or damaged mitochondria. To extend and better understand the functional roles and importance of migrasomes, it is first essential to well understand the basic molecular mechanisms behind their formation and function. Herein, we endeavor to provide a brief and up-to-date description of migrasome biogenesis, release, characterization, biological properties and functional activities in cell-to-cell communication, and we will discuss and propose putative new functions for these vesicles.

## Background

The importance of the extracellular vesicle (EV)-mediated intercellular communication is emerging at a fast pace. EVs are released under both normal and pathological conditions by exerting pleiotropic biological functions [1], and their presence has been observed in many body fluids [2, 3].

Although the term “extracellular vesicles” is currently used to refer generically to all heterogeneous secreted membrane-bound structures, EVs can be broadly divided into different categories, based on their own features: exosomes, ectosomes, oncosomes and apoptotic bodies. The serendipitous discovery of migrasomes, allowed Li Yu’s group to outline, in 2015, the origin of a vesicle unknown until that moment [4] that might join the aforementioned EVs’ list.

As it can be inferred by its name, a migrasome is a transient organelle produced on the tips or at the intersections of the long tubular and tiny protrusions, called retraction fibers (RFs), trailed behind migrating cells during a process defined as “migracytosis”. They are formed after the break of these filamentous structures and can brake, in order to release their cytoplasmic content in the extracellular space. Interestingly and alternatively, they can be collected by surrounding cells, and their cargo can be internalised.

The Yu group’s discovery primed researchers to unveil their biological role in cell-to-cell communication and in the maintenance of cellular homeostasis, a topic still highly debated and studied [5].

By this review, we summarize what is known, so far, about the nature and content of migrasomes, their role in different biological processes, and we discuss putative future research lines in this field, in both physiological and pathological contexts.

## The heterogeneous and dynamic world of extracellular vesicles

Intercellular communication is an essential hallmark of multi-cellular organisms mediated by direct cell–cell contacts or by transfer of secreted molecules. Indeed, mounting evidence is increasing on the extracellular vesicle (EV) release-mediated communication [6].

All cells are able of secreting different types of membrane vesicles, known as EVs, or nanoparticles named exomeres, and this is a widely evolutionary conserved process: from bacteria to humans and plants [7].

**Fig. 1:**
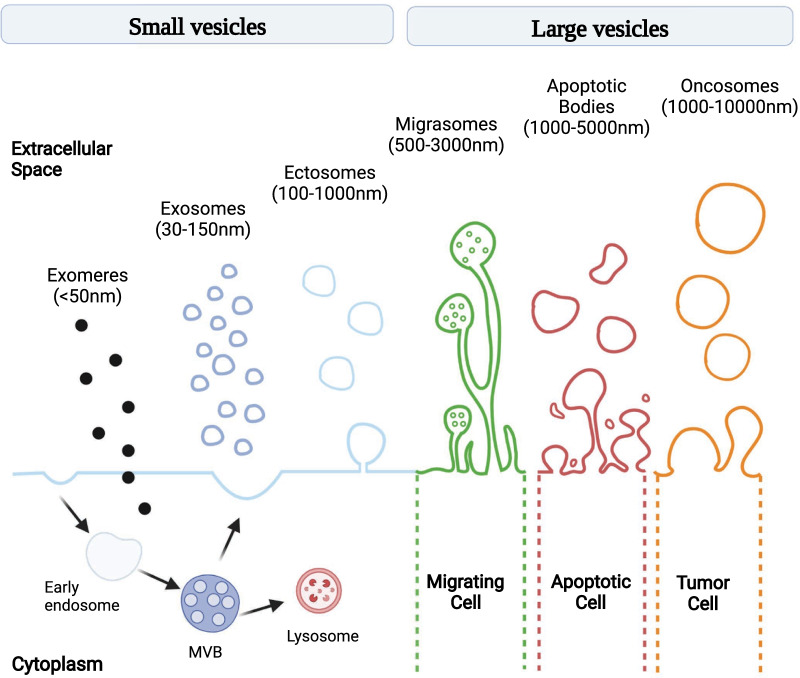
EVs Classification. Schematic illustration of EVs subtypes according to their different size and genesis model. Exomeres represent a population of non-membranous nanoparticles smaller than 50 nm with distinct proteomic signature (e.g. HSP90AB1, IDH1) and biodistribution. Exosomes are the smallest EV sub-population originally generated from an intracellular vesicle trafficking system, which involves biogenesis of early endosomes and multivesicular bodies (MVBs) following their fusion with the plasma membrane. Ectosomes are mid-sized EVs (100–1000 nm) released from cells via the outward budding and fission of the plasma membrane, transferring bioactive cargoes. Migrasomes (up to 3000 nm) were found to be released from the tip of retraction fibers, that cells leave behind as they migrate. They have been described as pomegranate-like structures, large vesicles encapsulating numerous smaller vesicles ranging 50–100 nm and their function remains to be better elucidated in upcoming investigations. Apoptotic bodies (1000–5000 nm) are irregularly shaped, nucleic acid-containing cell fragments released during the terminal stage of apoptosis. ABs are normally phagocytosed to maintain homeostasis but have also been reported to facilitate horizontal transfer of oncogenes in tumors. Oncosomes are large-sized EV subpopulations (1000–10000 nm) released from large protrusions of the plasma membrane during amoeboid migration of metastatic prostate cancer cells. Oncosomes have been reported to transfer functional miRNAs, mRNAs and proteins to promote cancer metastasis

Although the term “extracellular vesicles” is currently used to refer generically to all heterogeneous secreted vesicles, they can be broadly divided into different categories, based on their own features: exomeres, exosomes, ectosomes, migrasomes, apoptotic bodies and oncosomes (Fig. 1).

Exomeres represent an abundant population of non-membranous nanoparticles (~ 35 nm), enriched in proteins involved in metabolism including MAT1A, IDH1, GMPPB, UGP2, EXT1, PFKL and glycan processing factors (MAN2A1, HEXB, GANAB); this suggests that exomere cargos may mediate the targeting of recipient cells through specific glycan recognition, and modulate glycosylation in recipient cells [8].

It has been found that exomeres are selectively enriched in proteins associated with coagulation (e.g., Factors VIII and X) and hypoxia [9], as well as in proteins involved in metabolism, especially in “glycolysis” and the “mTORC1” metabolic pathways [10], with this suggesting their potential roles in influencing the metabolic program in target cells.

Exosomes are nano-sized EVs (30–150 nm) originating from the late endosomal trafficking machinery. They are gathered intracellularly into multivesicular bodies (MVBs) and ultimately released as a result of MVB fusion with the plasma membrane [11, 12]. An analysis of the proteins most frequently identified in exosomes highlights the presence of the tetraspanin family members CD9, CD63, CD81 and CD82, the small actin-binding protein Cofilin1, and heat shock proteins such as Hsp70-Hsp90, and Tsg10, flotillin, ALIX (protein regulating cellular mechanisms) [13].

Ectosomes, also reported as membrane particles, are cell surface derived EVs typically larger than exosomes (diameter: 100–1000 nm), originating from direct budding of the plasma membrane [14]. Compared with exosomes, ectosomes release from parent cells to the extracellular fluid in response to appropriate triggers is much faster: once specific molecules accumulate at plasma membrane microdomains, these vesicles generate and are promptly released [15].

In addition to the presence of integrins and tetraspanins, ectosomes contain other proteins, such as the matrix metalloproteinase MT1-MMP, two glycoprotein receptors (GP1b and GPIIb/GPIIa), and the adhesion protein P-selectin [16].

A peculiar type of large EVs are the interesting migration-related vesicles termed migrasomes (up to 3000 nm). They are oval-shaped vesicles formed during cell migration and contain, in turn, numerous smaller vesicles [17]. They represent the main focus of this review, and we will thus deeply turn on their discussion later on.

Apoptotic Bodies are particles of relatively large size (1-5 μm), released by tumor cells and other cell types, upon their triggered collapse that results in karyorrhexis (nuclear fragmentation), increase on membrane permeability and externalization of phosphatidylserine (PS) [18, 19].

These vesicles contain different components of dead cells [20], including proteins, lipids and nucleic acids, even in fragments.

The apoptotic bodies can act by “dispatching suicide notes” to the surrounding environment. Indeed, in early phases of apoptosis, their membranes display increased permeabilization, so allowing the release of proteins into the local microenvironment [21].

Large Oncosomes represent an additional class of tumor-derived EVs, so called because of an atypically large size (1–10 μm) and their abundant oncogenic cargo [22]. This EV population can propagate oncogenic information or signals, including transfer of signal transduction complexes, across tissues, originating directly from plasma membrane budding and expressing ARF6 [23]. Large oncosome formation is particularly evident in highly migratory and aggressive tumor cells from prostate, breast, bladder, lung cancers, with an amoeboid phenotype. They contain metalloproteinases, RNAs, caveolin-1, and the GTPase ADP-ribosylation factor 6 [24].

## Biorigin and architecture of migrasomes

In 1963, Taylor and Robbins performed a detailed light microscopy and TEM study, by which they observed the formation of long tubular structures, whose tips were characterized by some enlargements containing cytoplasmic granules, released by different types of migrating cells on the substratum [25]. They named these structures “retraction fibrils”, later renamed as “retraction fibers”. Until now, retraction fibers have received little attention despite their widespread presence in different cell types.

Recently, it has been described the biogenesis of a migration-dependent mechanism for releasing cellular contents called “migracytosis” [26], this originating from retraction fibers, indeed. Ma et al. found that, during migration, a cell is able to leave a new ring-like organelle, named migrasome [4], deriving from retraction fibers at the rear-edge of the migrating cell. Indeed, during migracytosis, RFs are pulled out from the trailing edge of a migrating cell. This means that RFs must adhere to the extracellular matrix (ECM). Of note, it has been clarified that integrins are gathered into puncta on RFs before migrasome formation, and that integrins-ECM interactions are necessary to establish the adhesion sites along RFs, which then act as platforms for migrasome biogenesis [27]. As the retraction fibers disintegrate, migrasomes filled with cytosolic material could be released and remain into the extracellular environment, to eventually and directly be taken up by surrounding cells.

By mass spectrometry analysis, it was elucidated how Tetraspanin-4 (TSPAN4), a Tetraspanin family member, plays a crucial role, being not merely a migrasome biomarker, but the main promoter of their production [4]. Indeed, TSPAN4 interacts on the membrane with cholesterol and integrins, in order to establish a Tetraspanin-enriched microdomain (TEM), also called Tetraspanin-web, thus ensuring the migrasome formation [28]. Tetraspanins are members of a family of small evolutionary conserved membrane surface proteins, characterized by four transmembrane domains and involved in the modulation of several different biological processes, such as cell signaling, trafficking, cell development, migration and cancer [29].

Beyond the conventional (and convenient) use of TSPAN4 for migrasome detection, recent evidence pointed out the adoption of different molecular probes and protein markers useful for their visualization, such as WGA (Wheat-germ agglutinin, a sialic acid and N-acetyl-D-glucosamine-binding lectin) or NDST1 (bifunctional heparan sulfate N-deacetylase/N-sulfotransferase 1), PIGK (phosphatidylinositol glycan anchor biosynthesis, class K), CPQ (carboxypeptidase Q), and EOGT (EGF domain-specific O-linked N-acetylglucosamine transferase) [30, 31].

At variance with other EVs, such as oncosomes, apoptotic bodies or exosomes, migrasomes are vesicles with a peculiar size (0.5–3 µm) and internal architecture (Table 1). Indeed, due to the presence of numerous intraluminal smaller vesicles, they could be named also pomegranate-like structures (PLS). Moreover, if exosomes or other vesicles are released after fusion of multivesicular bodies with plasma membrane, migracytosis involves the translocation of cytoplasmic material into migrasomes that are released after the breakage of the cell’s RFs [32].

**Table 1:** Characteristics of migrasomes

**MIGRASOMES FEATURES**	
**SIZE**	
0.5–3 μm	
MARKERs	
TSPAN4, Integrin 5α, CPQ, NDST1, PIGK, EOGT, WGA	
**CONTENTS**	
Chemokines, Cytokines, Growth factors, mRNAs, proteins, Damaged Mitochondria	
LIPIDS	
Cholesterol	
ORIGIN	
After the Retraction Fibers (RFs) break	
**MECHANISM OF RELEASE**	
Self organization of tetraspanins and cholesterol in macrodomains (TEMAs), during cell migration	
**DETECTION METHODS**	
Electron microscopy, WB for specific migrasomes markers, Immunofluorescence, Live-imaging	
**ISOLATION METHODS**	
Ultracentrifugation	
**REFERENCES**	
[4, 5, 7, 25, 27–31, 33–36, 36, 37, 43, 44]	

These vesicles have been found in many cell types including normal rat kidney epithelial cell line (NRK), mouse embryonic fibroblasts, mouse embryonic stem cells, human breast and colon cancer cells. Migrasomes are also distributed in various organs, such as human stroke specimens, mouse eye, rat lung, rat/mouse intestine. They could be present inside cavity such as pulmonary alveoli, blood vessels or lymph capillaries [33].

Migrasomes can be isolated from conditioned cell culture media or bodily fluids by concentration via ultracentrifugation and subsequent density-gradient purification. Although this method is widely used for migrasome fractionation, it implies the use of a high cell number. Moreover, since the final product of the isolation protocol results in a highly enriched rather than a “pure” migrasome sample, other quality control experiments are more suitable and used.

To overcome this pitfall, it has been recently demonstrated that the deployment of functional substrates modified with a mixture of cell-penetrating peptides, such as pVEC and R9, fosters cell mobility and increases migrasome outbreak from longer RFs. Furthermore, the designing of a vesicle-capturing interface covered by both cell-penetrating peptide and integrin-binding peptide (e.g. RGD) encourages cell body detachment after EDTA treatment [34]. This approach helps to reach a reliable migrasomes fraction, suitable for further analysis.

## Migrasome as a promoter of intercellular communication

**Fig. 2:**
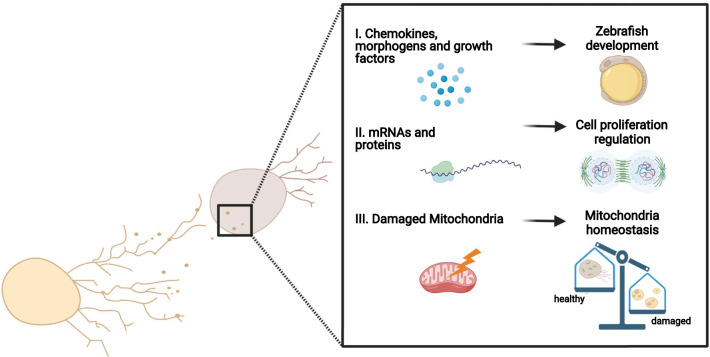
Migrasome biological functions. Schematics of migrasome content and putative roles of their uptake by migrating cell. The different contents of vesicles are illustrated in the right enlarged panel. So far, it is known that migrasomes could contain (I) chemokines or cytokines and morphogens to promote a correct zebrafish embryogenesis; (II) mRNAs and proteins involved in the cell proliferation modulation; (III) mild damaged mitochondria to ensure mitochondrial homeostasis within the cell

It has been found that migrasomes play a crucial role in many instances, from development to cell homeostasis (Fig. 2). For example, the formation of migrasomes on long cellular projections in zebrafish gastrulas has been reported [35].

Indeed, migrasomes released by mesoendodermal cells, enriched in the embryonic shield cavity, attract a particular subpopulation of cells named Dorsal Forerunner Cells (DFCs) at the edge of the shield through a CXCL12-mediated chemoattraction. Thus, being enriched of signaling molecules -including morphogens, growth factors and cytokines- migrasomes appear to be essential for the orchestration of embryonic organogenesis.

Interestingly, one cell’s migrasomes can be collected by surrounding cells, underlying the existence of a lateral transfer of cellular material. Indeed, it is not a long time since Yu’s group demonstrated that migrasomes contain proteins and mRNAs, such as the Pten full-length transcript and protein that can regulate Akt phosphorylation and, thus, proliferation in recipient cells [36].

Further, mitochondria homeostasis is essential for the proper biological functions (energy and aminoacids production; heme, and fatty acids synthesis), constantly maintained inside the cells. In fact, to ensure mitochondria stability and activity, cells have evolved several quality control processes, consisting of a finely tuned turnover of portions of the same mitochondria or damaged mito-proteins [5].

Thus, the removal of unfunctional mitochondrial parts, together with the production of new components during mitochondrial biosynthesis, represent the complemental mechanisms that ensure each cell optimal energetic and metabolic conditions.

Therefore, a new quality control mechanism, based on cell migration propensity, is now unveiled. Indeed, when cells migrate, migrasomes are released to mediate “mitocytosis”, a process consisting of recycling mild damaged mitochondria characterized by condensed matrix and swollen cristae. Since migration requires a higher energy demand, mitocytosis correlates migrasome production with mitochondrial homeostasis, to prevent an excessive stress due to the elevated respiration rate and the consequent increment of ROS levels.

## Clinical relevance of migrasomes function

Contextually to the aforementioned physiological implications of migrasomes in cell- to-cell communication and in the maintenance of cell homeostasis, the clinical application of migrasome modulation represents a path not well explored so far.

Following an ischemic injury of the central nervous system induced by a hight-salt diet, a reduction of microglia/macrophages was observed in association with enhanced migrasome production and inflammatory status [37]. Migrasomes released in the ischemic brain are mainly filled with metabolic enzymes, together with several cytoskeleton components, including contractile factors such as actin and myosin [33].

Two are the hypothesized roles for migrasomes detected in the brain parenchyma, as a consequence of such neuronal damage. On one side, the occurrence of migrasomes enriched of neuronal fragments near the shrunk neurons, and the correlation between migrasome formation and neuronal loss suggest that migrasome could aggravate neuronal shrinkage. On the other side, migrasomes might carry off fragments of damaged neurons acting as neuronal scavengers [37]. While migrasome’s contribution to neuronal damage is not yet completely unveiled, protective effects of exosomes against ischemic brain injury have been largely established [38]. Due to their own properties (e.g. low immunogenicity, long half-life and solid targeting capability), exosomes behave as a natural “shuttle system”, which can easily pass through the blood–brain barrier to deliver associated mRNAs, microRNAs and proteins. By providing these components, exosomes positively affect damaged and hypoxic brain microenvironment, promoting vascular remodeling and neurological functions [39], attenuating neuronal apoptosis [40] and inflammatory response [41].

More recently, it has been proposed to measure migrasomes as a useful biological diagnostic marker for the early renal system injury in diabetic-nephropathy patients. It was thus found that when podocytes undergo damages, they increase the amount of released migrasomes, compared to normal and functional podocytes. Indeed, injured podocytes take advantage of migrasome release to compensate for the loss of functional podocytes, rescuing a proper glomerular permeability [42].

Moreover, it has been demonstrated how podocyte-derived migrasomes differ from podocyte exosomes not only in size and content, but also for the mechanism and the circumstance of release. Indeed, the size of migrasomes (400–2000 nm) freed by podocytes is larger than that of podocyte-derived exosomes (100 nm). The respective content is also different: podocyte migrasomes and exosomes differ on the levels of different miRNAs (such as miR661, miR611, miR4286 or miR221-3p, to provide a few examples) [43]. Also, podocytes exosomes are stored in MVBs and released after their fusion to the plasma membrane, while podocytes migrasomes are released along the tiny and tubular structures pulled out from the cell’s surface.

Interestingly, also the conditions, during which these two types of EVs release occur, are different: while exosomes release by kidney tubular cells is not a cell-injury specific event, migrasomes can be truly cell damage-associated vesicles, since their generation is dependent on cell motility and, in podocytes, motility is closely associated with cell damage [42].

Altogether, these evidences highlight the importance of migrasomes also in the clinics.

## Putative roles of migrasomes and future perspectives

It is very recent the finding that migrasomes, a new type of extracellular vesicles whose clearest marker is Tetraspanin-4, can be released as small breadcrumbs by different migrating cells, and mediate cytoplasmic release. It was indeed demonstrated that migrasomes play a role in a new way of intercellular communication, since they are involved in some processes such as embryogenesis modulation and mitochondrial homeostasis [5, 35].

**Fig. 3:**
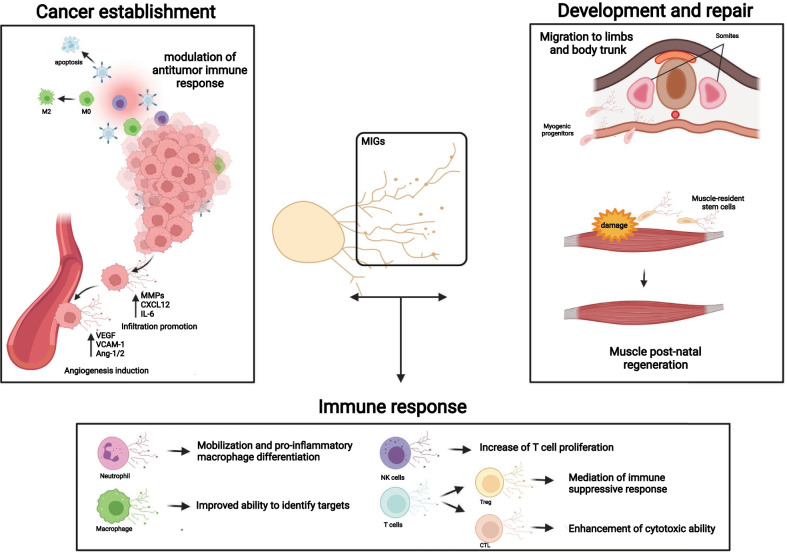
Migrasomes in different contexts. Schematic illustration of migrasomes putative roles in distinct scenarios. In TME cancer cells might release migrasomes to facilitate their intravasation, tissues infiltration and angiogenesis together with the enhancement of the immunosuppressive effects. During the skeletal muscle development, myogenic precursors-derived migrasomes could orchestrate a finely tuned motility of neighboring cells, thereby promoting a more concerted migration towards limbs and body trunk; while, after injury, the muscle-resident stem cells-derived migrasomes may be involved in a more rapid repair of the damage. According to the immune population type, migrasomes could exploit several crucial roles: from regulation, activation or mobilization of other immune cells (migrasomes released by the antigen presenting cells, for example), improvement of target identification (macrophage-derived migrasomes, full of processed antigens) to enhancement of cytotoxic/suppressive ability in an environmental-dependent manner (T cell-derived migrasomes)

Of note, it is widely known that migrasome release is a process underlying the ability of cells to migrate. Considering that there are contexts, in which migrating cells play a crucial role, such as cancer establishment and metastasis progression, cell development and the immune response, it would be very interesting to focus future investigations on those aspects (Fig. 3), with the aim of discovering further key roles for these vesicles.

Indeed, it is conceivable that migrasomes released by cancer cells may exacerbate tumor aggressiveness, accelerating cell proliferation or promoting metastasis formation.

To reduce mitosis duration, thereby increasing the rate of cell division, migrasomes could be enriched of mitogenic signals or factors. As cancer cells proliferate, they exhibit a higher demand of nutrients and oxygen that are supplied through blood vessels. Thus, given their marked propensity to invade surrounding tissues, heading towards the source of energy, tumor cells could release migrasomes enriched with metalloproteases or enzymes that regulate ECM stiffness, promoting a well-orchestrated infiltration process.

Tumor-derived migrasomes could be also evaluated as the containers of pro-angiogenic factors, such as vascular endothelial growth factor (VEGF) and angiopoietins (Ang-1/2) or suppressors of adhesion molecules such as E-selectins or vascular cell adhesion molecules-1 (VCAM-1).

Since it is strictly necessary for metastatic cells to avoid interactions with the immune system, migrasomes released in the tumor microenvironment could negatively modulate it, therefore encouraging cancer cells’ immune escaping. Indeed, they might induce apoptosis in effector T cells, reduce proliferation of Natural Killer (NK) cells or induce monocytes differentiation into immunosuppressive macrophages.

Cell migration is a key process also for cell development and repair. For example, during skeletal muscle development, myogenic progenitors could release migrasomes, during their migration towards prospective skeletal muscles of the trunk, with this representing an unveiled strategy for increasing their own motility.

Moreover, in chronic myopathies and in acute injuries, muscle-resident stem cells may release migrasomes, rich of contractile proteins or muscle growth factors, taking part to the post‐natal muscle regeneration by mediating new fibers formation, as previously reported for other types of EVs [44].

The immune system has evolved, over time, a highly controlled regulation and cooperation system consisting of different cellular (sub)types interacting each other to activate, inhibit, differentiate, and/or collaborate, to improve this fascinating protector machine. In order to fulfill this requirement, the immune system evolved in parallel the ability of using different types of communication to share or release information among immune cells. Some immune cell interplays happen over long distances, through the secretion and diffusion of soluble mediators, such as cytokines and chemokines, while others require T cells to come in close contact among them, or with other cell types (such as in the case of the immunological synapse formation with antigen presenting cells) [45].

Besides these canonical processes, it is also known that immune cells can communicate through alternative means, by the secretion of extracellular vesicles, such as endosome-derived exosomes [46]. Unlike those ones, released in an unoriented way in the extracellular space, migrasomes trails created by migrating cells could represent a more finely guided path for the oncoming cells, providing them with specific instructions and/or direction. In this way, the uptake of migrasomes may be directed to a limited niche of targeted cells, allowing them to distinguish and catch these vesicles.

Neutrophils and macrophages, that represent the first line of defense against the hexogen factors and pathogens, may also take advantage of migrasome release to carry out the innate immune response. Neutrophils might modulate innate surveillance stimulating a pro- or anti-inflammatory response, in an environmental-dependent manner, through cytokines or chemokines but also miRNAs, able to induce pro-inflammatory macrophages polarization. Macrophages, on their side, by their scavenger activity, could ingest and degrade foreign material, packing some of its components inside migrasomes. Thus, the release of these vesicles may educate neighbour cells to specifically identify the target to be destroyed. It could be possible that also NK cells, involved in early defense against tumors and infection, could release these vesicles, putatively full of miRNAs or cytotoxic proteins exerting antitumor effects, or that they could stimulate immune cells, by increasing T cell proliferation or stimulating monocytes.

The idea that T cells, so prone to migrate and invade tissues, might amplify and direct a massive migration through migrasomes release is plausible, too. Thus migrasomes, released during cell migration, would represent a driving cue to undertake the right direction for the oncoming cells.

Migrasomes released by this subset of immune cells, would thus perform a double and opposite function in (patho-)physiological contexts, by supporting lymphocyte recruitment, activation and cytotoxic ability, or by shut-downing the effective response through regulatory T cells (Tregs) suppressive signaling, also at inflammation sites.

In conclusion, the unique properties of migrasomes make them promising key players in different physiological phenomena, and their characterization has just started with a plethora of open questions awaiting answers, with the final promise to use migrasomes as a diagnostic marker or therapeutic tool.

## Data Availability

Not applicable.

## Abbreviations

ANG-1/2: Angiopoietin-1/2
CPQ: Carboxypeptidase Q
CXCL12: C-X-C Motif Chemokine Ligand 12
DFC: Dorsal forerunner cell
ECM: Extracellular Matrix
EDTA: Ethylenediaminetetraacetic acid
EOGT: EGF domain-specific O-linked N-acetylglucosamine transferase
EV: Extracellular Vesicle
MVB: Multivesicular body
NDST1: Bifunctional heparan sulfate N-deacetylase/N-sulfotransferase 1
NK: Natural Killer cell
NRK: Normal Rat Kidney epithelial cell line
PLS: Pomegranate-like structures
PIGK: PIGK (phosphatidylinositol glycan anchor biosynthesis, class K)
RF: Retraction Fiber
ROS: Reactive Oxygen Species
TEM: Tetraspanins-enriched microdomain
TEM: Transmission electron microscopy
TME: Tumor microenvironment
Treg: Regulatory T cell
TSPAN4: Tetraspanin-4
VCAM-1: Vascular cell adhesion molecule-1
VEGF: Vascular endothelial growth factor
WGA: Wheat-germ agglutinin
